# Statistical analysis of the community lockdown for COVID-19 pandemic

**DOI:** 10.1007/s10489-021-02615-9

**Published:** 2021-07-07

**Authors:** Shaocong Wu, Xiaolong Wang, Jingyong Su

**Affiliations:** 1 School of Computer Science and Technology, Harbin Institute of Technology, Shenzhen, 518055 China; 2 Shenzhen Key Laboratory of Visual Object Detection and Recognition, Harbin Institute of Technology, Shenzhen, 518055 China

**Keywords:** COVID-19, Agent-based simulation, Epidemic process analysis, Lockdown

## Abstract

As the global pandemic of the COVID-19 continues, the statistical modeling and analysis of the spreading process of COVID-19 have attracted widespread attention. Various propagation simulation models have been proposed to predict the spread of the epidemic and the effectiveness of related control measures. These models play an indispensable role in understanding the complex dynamic situation of the epidemic. Most existing work studies the spread of epidemic at two levels including population and agent. However, there is no comprehensive statistical analysis of community lockdown measures and corresponding control effects. This paper performs a statistical analysis of the effectiveness of community lockdown based on the Agent-Level Pandemic Simulation (ALPS) model. We propose a statistical model to analyze multiple variables affecting the COVID-19 pandemic, which include the timings of implementing and lifting lockdown, the crowd mobility, and other factors. Specifically, a motion model followed by ALPS and related basic assumptions is discussed first. Then the model has been evaluated using the real data of COVID-19. The simulation study and comparison with real data have validated the effectiveness of our model.

## Introduction

Since January 2020, the Lancet have published many papers and reviews that have revealed the high mortality rate and person-to-person transmission characteristics of the new coronavirus infection [6, 14]. As the global pandemic of COVID-19 is still happening around the world, it is vital to prevent the spread of the epidemic by implementing control measures [29]. There are many types of control measures, including community blockade, wearing masks, body temperature checking, access restrictions, traffic control, real names and ID numbers for censorship and surveillance, and so on. However, there is no consensus on the effectiveness of these measures. From the perspective of the growth rate of the epidemic, Wang et al. evaluated the effectiveness of control measures by establishing a dynamic model of development of the epidemic [30]. They pointed out that if the government did not take control measures, as of February 18, 2020, the number of existing infected people across the country would reach 16.98 million to 76.11 million. If the control measures are postponed one day, the number of existing infected persons will reach 27,000 higher than the actual number. If the control measures are postponed one week, the number of existing infected persons would be 785,000 higher. Maier et al. [22] further confirmed that control measures could change the exponential growth rate of the development of the epidemic significantly. Hernandez et al. [12] used ARIMA models and polynomial functions for analyzing the relation between COVID-19 behavior and population in a region. Research on the spread of COVID-19 is a global task. Catelli et al. [5] proposed a multi-lingual system in response to the low-resource language scenario. Ma et al. [21] built an artificial city in a simulated environment based on the ACP method, analyzed the impact on the development of the epidemic when implementing different control measures for different places, and pointed out that inspection and quarantine and self-protection can only delay the arrival of the peak of infection. A complete blockade is the only way to stop the spread of the epidemic. However, current research on the effectiveness of the control measures most focuses on the macroscopic growth situation in large regions and even the entire country. There are three types of epidemic models below:

- **Compartmental Models**. Compartmental Models divide the total population into different warehouses according to their different states. The process of epidemic spreading is abstracted as the population movements between different warehouses. Such models include the SIR model proposed by Kermack and McKendrick [19], and the SI model, the SIS model, the SEIR model, and so on [24].
- **Logistic Growth Models**. Logistic Growth Models were originally used to simulate population growth in a limited environment. They were subsequently applied in the simulation of infectious disease epidemic dynamics [20].
- **Network Models**. In Network Models, network nodes are used to represents in the population, and the network structure interconnections are used to simulate the relationship between individuals. Network models were used to study the law of the spread of an epidemic in a population [22].

Although these models are applicable to assess the macro situation of the epidemic, lacking the accurate description of the state of each individual in the population makes these models unsuitable for the accurate simulation of the spread of an epidemic in the community. There is no effective method for fine-grained evaluation of control measures in small areas such as residential communities. It is important to simulate the spread of the epidemic at the community level. But there is a class of epidemic models that offers the possibility of overcoming this limitation, called Agent-based Models. Agent-based models track the spread of epidemic at the individual level and simulate the complex interaction factors between individuals. Among them are the ALPS model [26] and BioWar model [4].

This paper studies real data of COVID-19 and proposes a model based on the ALPS to simulate the spread of the epidemic in the community. We have performed a quantitative evaluation of the effectiveness of the proposed model. The rest of this paper is organized as follows. Section 2 introduces the underlying assumptions and motion models of the ALPS model first. The COVID19_ALPS model and its implementation details are proposed subsequently. Section 3 presents the simulation results of the COVID19_ALPS model under different experimental settings and evaluates the comparison with real data from Wuhan, China. The paper ends by summarizing the advantages of the proposed model and suggesting improvements in the future.

## The COVID19_ALPS model

### Related work

Hunter et al. [15] believed that the models relating to the spread of epidemics through human contacts can be broadly categorized into two main classes, population-level coarse modeling and agent-level modeling. Compartmental models and network models are the representatives of the former. The population-level coarse modeling sets consistent parameters for all individuals in the same population, which is a typical top-down method. While the population level dynamical evolutions of population variables are simple and very effective for the overall assessment, they do not take into account any social dynamics, human behavior, government-mandated restrictions, and complexities of human interactions explicitly.

Agent-based modeling is a bottom-up approach, which is the opposite of Population-level coarse modeling. Agents are artificial individuals programmed to perform pre-defined operations. They operate based on their behavior, interact with the environment, collaborate or compete with each other agents. With this characteristic, it is possible to visualize the behaviors of agents as a physical system, such as simulations of evacuations, traffic, biological systems, infections, etc [7]. Therefore, we can treat the individual as an agent in the simulation. The models that study these human-level factors and variables while tracking disease at an individual level are called agent-based models. The advantages are that they are more detailed, and one can vary the parameters of restriction measures, such as social distancing, at a granular level to infer overall outcomes [16].

Agent-based models have wide applications in the transmission of epidemics, but these models have different implementation details. This phenomenon means that many agent-based models are fundamentally different. We collect some representative references, as shown in Table 1. The characteristic information in each reference is presented separately, including authors and date (A & D), the interaction between agents (Interaction), the number of agent states (States), focus on the epidemic (Epidemic), and the simulated environment (Environment).

**Table 1:** The characteristic information in related references

A & D	Interaction	States	Epidemic	Environment
Shamil et al. 2021 [23]	Event-driven	5	COVID-19	A scaled-down version of New York
Erik Cuevas. 2020 [9]	Motion Model	2	COVID-19	Simulated facilities
Petrônio et al. 2020 [25]	Motion Model	4	COVID-19	Simulated society
Alzu’bi et al. 2021 [1]	Motion Model	3	COVID-19	Simulated urban area
Takeshi et al. 2021 [17]	Cellular automaton model	6	COVID-19	Simulated Social Network
Nicolas et al. 2020 [13]	Contact matrix	Unknown	COVID-19	Simulated society
Thomas et al. 2021 [11]	Motion Model	2	COVID-19	Simulated “supermarket” scenario
Dmytro et al. 2019 [8]	Event-driven	6	Syphilis	NetLogo [28]
Parastu et al. 2013 [18]	Contact network in 3 levels	5	Tuberculosis	Simulated Social Network
Enrique et al. 2011 [10]	Individual mobility patterns	4	H1N1	Simulated Social Network
Georgiy et al. 2007 [3]	The probabilistic network and air travel	4	No specific goal	homogeneously mixed groups
Eyob et al. 2004 [27]	Agent-communication language	4	HIV	Environment agent

From these references, We have summarized the following key points:

- **Most Agent-based models divide agent states based on the compartmental model** [1, 3, 9, 10, 25]. Compartmental models such as SI, SIR, and SEIR divide the population into fixed categories, including Susceptible(S), Exposed(E), Infected(I), and Removed(R). This makes agent states simple and clear enough to avoid the uncertain factors of the fuzzy state. However, simple state division may not be sufficient to meet the requirements of accurate simulation. Some studies use more complex state division [17, 18, 27].
- **There are different definitions of “agent” in these studies.** Most researchers regard individuals as agents, and agents are only a kind of representative of humans in the simulation model. However, other researchers have adopted a broader definition because they believe that agents can represent any element in the simulation model, including environment [25], experimental controller [27], and statistical analyzer [27].
- **“Epidemic” and “Interaction” are significantly related**. COVID-19, H1N1, and tuberculosis are airborne infections. Therefore, the model for studying these diseases uses the Contact network, mobile model, and behavior model as the interaction between agents. These interactions are based on the agent’s movement in the simulated environment, which is conducive to calculating the distance between the agents to determine whether it is infected. HIV and syphilis are spread through sexual activity. At this time, it is meaningless to calculate the spatial distance between agents. The most important thing is to judge whether they have intimacy, skin contact, or blood contact through social events.
- **The simulated environment is diverse**. The scale of the simulated environment can be divided into three levels: facilities, urban area, and society. These environments are represented by two-dimensional spaces with clear boundaries, length, and width. In addition, there is a special kind of environment called social networks. The social network model does not calculate the agent’s movement but simulates the agent’s interaction through the links between network nodes.
- **Data-driven is the leading cause of the difference**. Researchers used different data in different studies. Models are data-sensitive and different data leads to diverse structures. Objectively, this makes data-driven models extremely demanding on the data used. For example, in reference [23], the authors suggest that their work requires four types of data, including location-specific data, physiological data, the data related to COVID-19 disease, and smartphone-related statistics. However, other studies use completely different data, such as Geographic Information Systems (GIS) [13], call detail records [10], land use statistics, and Gross Domestic Product (GDP) [25].
- **Metrics reflect different research purposes**. Although many researchers use agent-based methods to study COVID-19, their concerns are not the same. The most common metrics in these studies are infection rate, recovery rate, and mortality rate. On the other hand, some researchers have focused on the intensive care unit (ICU)-bed occupancy [13] and economic activities [17].

These studies have proved the effectiveness of agent-based modeling methods in disease transmission, which laid the foundation for our research on community lockdown.

### Agent-level pandemic simulation

**Fig. 1:**
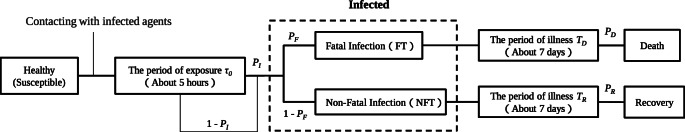
The course constructed by ALPS

Srivastava [26] proposed a method called Agent-Level Pandemic Simulation (ALPS) for simulating the spread of the infection in the community. The course constructed by ALPS is shown in Fig. 1. Healthy agents will enter the period of the exposure after contacting with an infected agent. When the cumulative time of exposure reaches *τ*_0_, healthy agents will become infected with *P*_*I*_. This infection is divided into Fatal Infection (FT) and Non-Fatal Infection (NFT). The probability of an infected agent becoming FT is set as *P*_*F*_. Then, these infected agents will enter the period of illness. When the cumulative time in this period reaches *T*_*D*_ or *T*_*R*_, according to the infection type, they will enter the stage of death and recovery with *P*_*D*_ or *P*_*R*_ respectively.

There is two part of the construction of the ALPS model:

1. The construction of a motion model for every agent.
2. The underlying assumptions of simulation.

For every individual’s motion, ALPS constructed the motion model as follows.

1 $$ v_{i}\left( t \right) =\mu v_{i}\left( t-1 \right) +\left( 1-\mu \right) \alpha \left( h_{i}-x_{i}\left( t-1 \right) \right) +\sigma w_{i}\left( t \right)  $$

2 $$ x_{i}\left( t \right) =x_{i}\left( t-1 \right) +\delta v_{i}\left( t \right)  $$

Equation (1) defines a resident individual *i* with an instantaneous speed *v*_*i*_(*t*) at moment *t*. The speed *v*_*i*_(*t*) is determined by the weighted sum of the following three components:

- $ \mu v_{i}(t-1) (0\leqslant \mu \leqslant 1)$: *v*_*i*_(*t* − 1) is the speed of the previous moment. The parameter *μ* quantifies the degree of willingness that resident individual follows the lockdown policy and stays in residence. *μ* = 0 means the individual resident returns to the residence, follows the lockdown policy and stays in residence. *μ* = 1 means the community does not take the lockdown or the resident individual does not obey the lockdown and keeps moving freely.
- $\left (1-\mu \right ) \alpha \left (h_{i}-x_{i}\left (t-1 \right ) \right )$: the velocity component, which guides individual resident return to the residence. $ \alpha \in \mathbb {R}_{+} $ here denotes the speed of the resident individual returning to the residence.
- $ \sigma w_{i}\left (t \right ) $: independent Gaussian increment, where $ w_{i}\left (t \right ) \sim {\mathscr{N}}\left (0,1 \right ) $. The position *x*_*i*_(*t*) of individual *i* at moment *t* is determined by both the position of the previous moment and the velocity of the current moment *v*_*i*_(*t*).

Once the motion model has been defined, some assumptions need to be specified based on real situations as follows:

#### The residential community is located in a square area *D*, where there are *h* units of buildings for residents to live in and these units of buildings are distributed in an equally spaced rectangular grid

There are total N resident individuals in the community. The simulation model is updated every time interval *t* (which is defined in hours).

#### Independent resident individual

Each resident has an individual motion model and probability of being infected. When the distance between a healthy resident and an infected person is less than *τ*_0_, there would be a risk of contact infection.

#### Unrestricted mobility without lockdown

Each individual can move freely, which is not affected by their age, gender or health problems. The factors of day and night are ignored. Residents in the community move freely at any time.

#### Mandatory homestay

If the community starts to implement the lockdown control on day *T*_0_, most residents will return to their residences within a few hours and stay in their residential units until the lockdown control ends (on day *T*_1_). Nevertheless, a small part of people (ratio set as *ρ*_0_) could still move freely after the lockdown. They are community service personnel and family members who purchase supplies or people who accidentally violate lockdown.

#### Sealed region boundaries

To avoid introducing complex multi-regional crowd interaction and transportation in the model, it is assumed that there is no situation where residents enter or exit the community. That is no inflow of outside population and no outflow of the population in the community. At the same time, the lockdown community boundaries are reflective. Residents cannot stay at the boundaries for a long time. They will be “persuaded to return” to make subsequent movement away from the boundaries when residents move near the community boundaries.

#### Fixed domicile

A certain number of unit buildings will be placed in the entire community. These unit buildings are distributed in a uniformly spaced grid. Every resident has a fixed living place, and the unit building that the resident belongs to will not change during the simulation process. When the lockdown is implemented, residents will return to their respective unit buildings. However, residents in the same unit building are not completely isolated in their residences, and they can contact each other to a certain extent, which makes it possible that infection would spread between different families living in the same unit or between family members in a single family.

#### No multiple infections or recurrence

Assuming that a resident could recover from the epidemic, the resident would not be infected by the same epidemic again, and there is no recurrence of the epidemic. At present, whether there is a secondary infection in COVID-19 is still a key issue to be solved, but some researchs have pointed out that there is no recurrence and secondary infection in COVD-19 [2], which make this assumption valid.

#### Single initial infection

The epidemic is often spread among the population from a single carrier of the virus. Assuming that the epidemic in the community is spread from an infected resident, the initial infection is randomly selected from the residents.

#### Constant immunity level

It is assuming that the probability of resident individuals being infected with the epidemic remains the same in the case of exposure. The immunity level of resident individuals would not increase or

decrease over time. All the resident individuals have the same level of immunity for the same epidemic, which means the same infection probability, recovery probability, and lethality probability among residents.

### The COVID19_ALPS model

The ALPS model provides an effective way to evaluate the spread of infection in local areas and the effectiveness of the corresponding lockdown. Srivastava has shown that implementing the lockdown in time would help reduce the highest infection rate and mortality rate in the community [26]. At the same time, the duration of lockdown and the timing of lifting lockdown also have a profound impact on the spread of the epidemic. On the other hand, we realize that some parameters and assumptions in the ALPS are common settings, which do not agree with the real situation of COVID-19. Therefore, we propose a COVID19_ALPS model based on the ALPS and the real data of COVID-19.

**Fig. 2:**
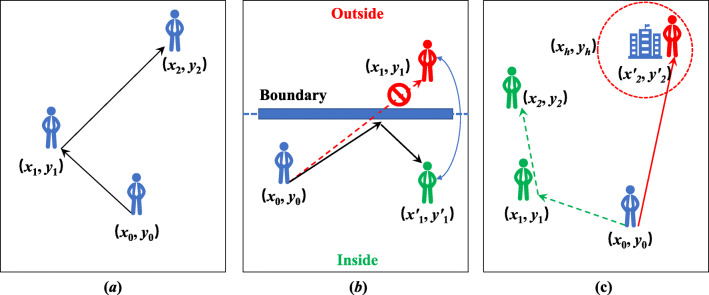
Modes of movement in COVID19_ALPS model. **a** Random movement; **b** Movement near the boundary; **c** Timely response to the lockdown

Agent (not dead) has three modes of movement in the COVID19_ALPS model shown in Fig. 2.

1. **Random movement**. Agents can move freely without restriction. The direction and speed of movement are determined by (1).
2. **Movement near the boundary**. In our simulation, the boundary is sealed, and no agent is allowed to leave. However, when the agent is near the boundary, the new position of the agent may appear outside the boundary after random movement. For example, the position of an agent at time *t*_0_ is (*x*_0_,*y*_0_) (inside). It will arrive at the position (*x*_1_,*y*_1_) (outside) at time *t*_1_ by random movement. It is necessary to reflect the moving path with the boundary as a mirror and generate a new position ((*x*1′,*y*1′) inside the boundary.
3. **Timely response to lockdown**. The agent moves randomly without lockdown (the green moving path). When the community is required to lockdown, the agent must immediately move straight to its houseunit (the red moving path). The longer the distance from the houseunit, the faster the agent moves. This process ends until the agent returns to the houseunit and starts the mandatory homestay.

**Fig. 3:**
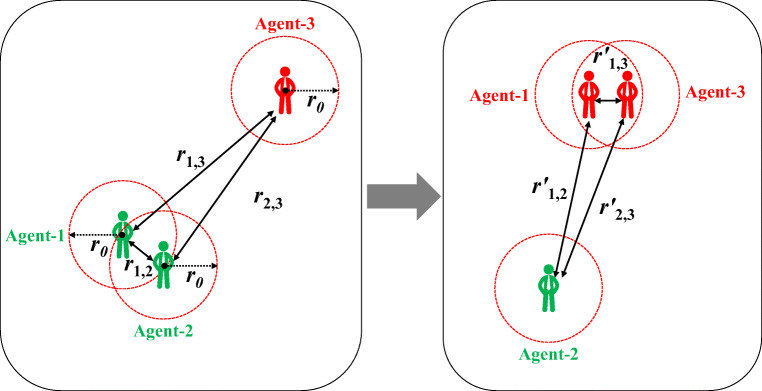
Example of the spread of COVID-19 between agents

In our model, the spread of COVID-19 among agents is shown in Fig. 3. COVID-19 spreads through close contact (the distance between two agents is less than *r*_0_). For example, at time *t*, agent-1 and agent-2 are healthy, and agent-3 is infected. Although the distance *r*_1,2_ between agent-1 and agent-2 is smaller than *r*_0_, the spread will not occur between the healthy agents. Both *r*_1,3_ and *r*_2,3_ are greater than *r*_0_, and there is no risk of being infected by agent-3. After the agents move, their new positions are as shown on the right. The distance *r*1,3′ between agent-1 and agent-3 is less than *r*_0_, agent-1 is infected by agent-3 with probability *P*_*I*_.

In addition, other contributions of our research are described as follows:

#### The reconstruction of the course consistent with COVID-19

**Fig. 4:**
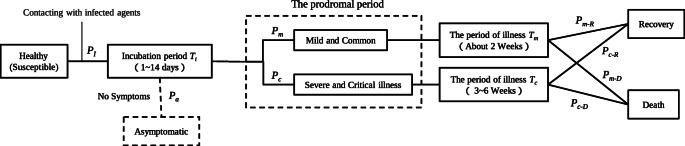
Improved course based on statistical data of COVID-19

The course of the disease shown in Fig. 1 is a general infectious disease course constructed by ALPS. It accumulates the contact time to make sure the agents are infected and determines the rehabilitation or death by distinguishing different types of infection. However, the setting of this course is not consistent with the actual situation of COVID-19 patients. This study resets the course of patients in ALPS, shown in Fig. 4, according to the relevant statistics of COVID-19 from *China-WHO Joint Investigation Report on Novel Coronavirus Pneumonia (COVID-19)*^1^ (hereinafter referred to as the investigation report).

#### More precise agent states settings

Existing work divides agents into Susceptible(S), Exposed(E), Infected(I) and Removed(R), which loses the details of COVID-19. In reality, COVID-19 patients will experience a variety of intermediate states in the process, from health to recovery/death. There are seven states in our proposed model: Healthy (Susceptible), Incubation, Asymptomatic, Mild and Common, Severe and Critical, Recovery and Death. These states setting is more in line with the real situation of COVID-19.

#### More reasonable exposure-infection model

Existing work believes that an agent’s infection depends on the level of exposure to another infected person. The amount of exposure in terms of the number of time units should be at least *τ*_0_. This means that sufficient exposure time must be accumulated to determine whether the agent is infected, but it is contrary to the real situation. In fact, the agent is not immune even if the exposure time is insufficient. In our proposed model, the susceptible agent will be infected with probability *P*_*I*_ at every moment *t* as long as it is in close contact with the infected agent.

Healthy agents are infected with probability *P*_*I*_ after contacting with infected persons and enter the incubation period *T*_*i*_. The incubation period is followed by the prodromal period, and agents will become mild and common patients or patients with a severe and critical illness. Finally, they are cured or died after the period of illness.

#### Reasonable parameter setting based on COVID-19 statistics

The setting of parameters related to the course is impractical in the ALPS model. It is used as a demonstration. This paper focuses on the COVID-19 pandemic in China. Due to the large differences of related data between provinces and cities in various periods in China, the relevant parameters are set based on the overall statistical mean. The parameters in our model are set based on statistics released by the authority, such as National Health Commission of China^2^ and WHO COVID-19 technical guidelines.^3^ The details of the parameters will be explained in Section 3.

#### Thorough study on the community scale

Srivastava has used a square domain with size 2 miles × 2 miles for a community in all experiments. The community scale is an unconsidered factor in the ALPS model. However, we believe that the impact of community scale cannot be ignored and make a thorough study on it. According to the national standard (*Urban Residential District Planning and Design Standard GB50180-2018*)^4^ published by the Ministry of Housing and Urban-Rural Development of the People’s Republic of China in July 2018, the scale of urban communities and the corresponding information are shown in Table 2. In order to facilitate subsequent model calculations, the relevant parameters here are all calculated as minimum values. Since the specific number of residential units in the community is not given, this study assumes each residential unit is 10 stories high, 2 households on each floor, and 3 persons per household. The number of residential units in the community is obtained according to the total population in the community. In order to facilitate the evenly distributed processing of the residential units in the simulation, the nearest perfect square number is taken as the number of residential units. Due to the large scale of the 15-minute pedestrian-scale community whose simulation requires a lot of computational resources, it will not be analyzed in this study for the time being. Relevant experiments are carried out around 10-minute pedestrian-scale communities, 5-minute pedestrian-scale communities, and neighborhood block.

**Table 2:** The size of urban community and its population in China

Community scale	15-min pedestrian-scale	10-min pedestrian-scale	5-min pedestrian-scale	Neighborhood block
Total population	50,000	15,000	5,000	1,000
The number of buildings	841	256	81	16
Community coverage area (*m*^2^)	3,140,000	785,000	282,600	20.000

## Simulation study and comparison with real data

In this section, we evaluate the proposed model under three cases: no lockdown, lockdown without lifting, and lockdown with lifting after a certain period of time. We simulate under the model by 50 runs. In each run, initial positions and motion states of residents are random. Furthermore, there is only one person initially infected. Each simulation runs for 100 days, and the model is updated iteratively in hours. Average results of 50 runs are reported.

### Experiment parameters

In this section, we will describe the meaning and value of each parameter shown in Fig. 4. The values of all parameters are chosen based on the epidemic data released by the authority.

- *P*_*I*_ is the probability of being infected after close contact with infected agents, with a value of 0.01 0.05.
- The proportion of mild and common patients *P*_*m*_ is 80%. The proportion of severe and critically ill patients *P*_*c*_ is 19.9%. But the value of the proportion of asymptomatic patients *P*_*a*_ is controversial. According to the information disclosed by the National Health Commission of China, the nationwide proportion of asymptomatic patients is 1.83%. However, the information published in the investigation report indicates that the value of *P*_*a*_ is 0.1%. The high degree of concealment of asymptomatic patients makes it difficult to be counted accurately. To avoid the uncertainty caused by this situation, we set *P*_*a*_ as 0.1% in this study. Asymptomatic patients are considered as a type of patients who are infectious but will not be detected and recover after 2 weeks.
- After the period of illness, the probability that mild and common patients to be cured *P*_*m*−*R*_ is set as 0.95, and the corresponding death probability *P*_*m*−*D*_ is 0.05. The probability that severe and critically ill patients being cured *P*_*c*−*R*_ is set as 0.86, and the corresponding death probability *P*_*c*−*D*_ is 0.14.
- According to WHO COVID-19 technical guidelines, the main transmissions of the COVID-19 are contact transmission, pollutant transmission, and airborne transmission, and so on. Normally, when a healthy individual and an infected individual are within 1 meter, they are considered close contact. Therefore, the maximum distance to catch infection *r*_0_ is set as 1 meter in this study.
- Following the underlying assumptions in the ALPS model shown above.

### Exemplar scenarios

We illustrate the use of the COVID19_ALPS model by showing its outcomes under three typical scenarios. In these scenarios, we set the number of agents *N* = 1000, houseunits *h* = 16 (4 ∗ 4 evenly distributed), and the size of the community is 140 ∗ 140. The total simulation time T is 100 days. We show the distribution of agents at the beginning, the 10^*th*^ day, the 20^*th*^ day, the 40^*th*^ day, the 80^*th*^ day, and the end. The blue dots represent susceptible agents, and the red dots denote infected agents. Recovery corresponds to the green dot, with death corresponds to the black dot. The black diamonds are houseunits. There is only one initial infection in all scenarios, which is highlighted by a red circle.

#### Without lockdown

**Fig. 5:**
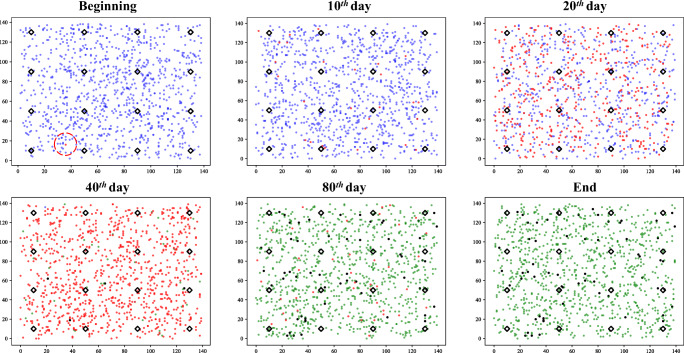
Scenario 1: without lockdown

COVID-19 spreads rapidly without any restraints as Fig. 5. There are almost no uninfected agents on the 40^*th*^ day. At this time, some infected agents have recovered or died. This is the worst case for the spread of COVID-19 that all agents are infected.

#### Lockdown and lifted restrictions

**Fig. 6:**
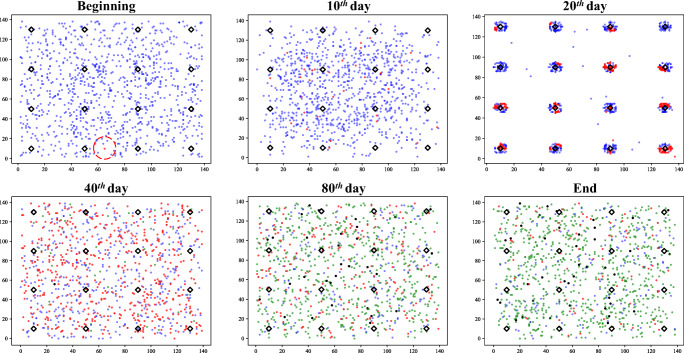
Scenario 2: Lockdown and lifted restrictions

(shown in Fig. 6). The timing of lockdown *T*_0_ is the 10^*th*^ day. Therefore, we can see that on the 20^*th*^ day, most of the agents stay in their houseunit, but some are still moving freely. Then the lockdown was lifted on the 30^*th*^ day (*T*_1_). This caused all agents to continue to move in the community, but it should be noted that the temporary lockdown significantly delayed the spread of COVID-19. The obvious evidence is that there are still some susceptible agents at the end of the simulation, and cause fewer deaths.

#### Lockdown until the end of simulation

**Fig. 7:**
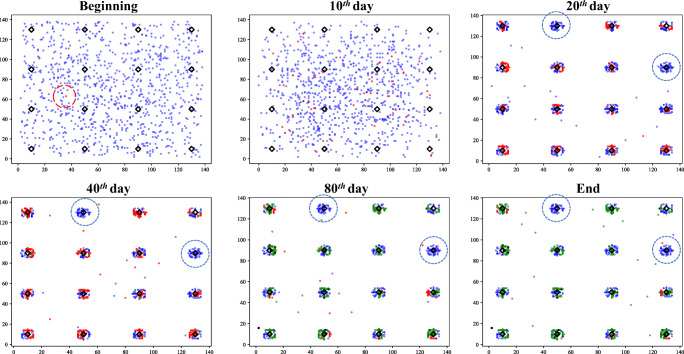
Scenario 3: Lockdown until the end of simulation

(shown in Fig. 7). In this scenario, the lockdown is also performed on the 10^*th*^ day. After that, all agents are required to return to the houseunit and stop moving. COVID-19 has spread freely in this community for ten days, which has caused infected agents to stay in each houseunit. Nevertheless, there are two special houseunits (highlighted by blue circle) with no infection until the end of the simulation. The compulsory homestay also restricts the spread of COVID-19 in the houseunit.

We will show more statistical details about these scenarios in Sections 3.3 and Section 3.4.

### The impact of residents’ moving speed and community scale on the spread of COVID-19 without lockdown

The moving speed of residents is determined by the motion model described by (1). This study evaluates the impact of residents’ moving speed and community scale on the spread of COVID-19 through four indicators:

1. *T*_*i*_: the time to reach the maximum of infected agents.
2. *P*_*i*_: the highest proportion of infected agents.
3. *P*_*d*_: the proportion of deaths when the simulation ends.
4. *P*_*r*_: the proportion of recovered agents when the simulation ends.

The parameter *α* in (1) is supposed to be 0.2, and the parameter *δ* is 1. Only by changing the parameter *σ* to control the moving speed of residents. The larger the value of *σ*, the faster the moving speed. The experiment results are shown in Table 2. When the community is set as a 10-minute pedestrian-scale community, due to the large scale of the community, the spread of COVID-19 has not ended until the end of the simulation. Instead, the results at the end of the simulation are recorded.

From the results in Table 3, we find that with the increase of moving speed, *T*_*i*_ will decrease gradually. The faster people move, the faster the COVID-19 spread. *P*_*i*_ and *P*_*d*_ are also at a higher level. Although there is only one infected person initially, the infections will reach the maximum after 35 to 45 days without any lockdown. After 100 days, about 5% to 6% of the community population will die of infecting the COVID-19. It should be noted that as the simulation enter the middle stage or late stage, most of the infected people have healed or passed away. However, a small amount of healthy agents just begin to be infected during these stages. Although the maximum proportion of infection is about 98%. In fact, there are no uninfected residents in the community at the end of the simulation. From the perspective of the entire simulation, the infection rate can be approximated as 100%.

**Table 3:** The impact of speed of movement and community size on the spread of the COVID-19

Community size and speed of movement	*σ*	*T*_*i*_(days)	*P*_*i*_(%)	*P*_*r*_(%)	*P*_*d*_(%)
Neighborhood block	5	39	98.20	94.10	5.80
	10	38	98.30	94.00	6.00
	15	31	98.70	93.40	6.60
5-minute pedestrian-scale	5	46	98.36	94.10	5.16
	10	42	98.74	94.34	5.56
	15	36	98.44	94.60	5.40
10-minute pedestrian-scale	5	-	19.20	2.12	0.12
	10	-	21.47	2.19	0.16
	15	-	23.37	2.23	0.19

Without the lockdown, the scale of the community has no significant impact on the spread of COVID-19. At the same speed, the larger the community, the longer it will take for the COVID-19 to spread. This characteristic means that COVID-19 break out in small areas is more rapid and difficult to control. In contrast, it will take longer for COVID-19 to spread in large and medium-sized areas before having the base of the outbreak. It will provide a rare opportunity for the implementation of anti-epidemic measures.

### The effectiveness of lockdown in restraining the spread of the COVID-19

In this section, we analyze the effect of lockdown in suppressing the spread of the COVID-19. The scale of the community chosen is neighborhood block and 5-minute pedestrian-scale community. The residents’ motion parameters *α*, *δ*, and *σ* are set respectively as 0.2, 1, and 10.

#### The impact of the timing of lockdown measures implementation (*T*_0_) on the control effect

**Fig. 8:**
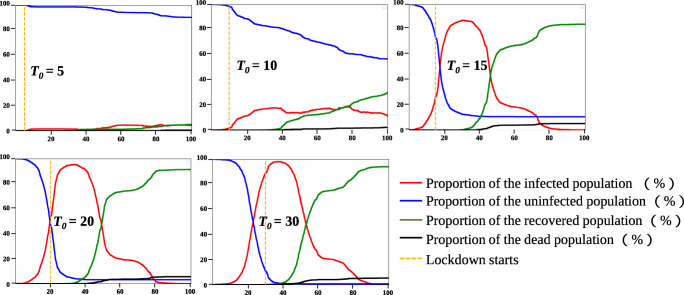
Schematic diagram of COVID-19 spreads in the community when the lockdown is implemented on the 5^*th*^ day, 10^*th*^ day, 15^*th*^ day, 20^*th*^ day and 30^*th*^ day. *T*_0_ is the time when the lockdown starts

In the simulation experiment, the start of lockdown is set as day *T*_0_. Once the lockdown begins, most residents will return to their respective residential units and would not leave the residential units until the end of the simulation. At the same time, few random resident individuals in the community continue to move freely regardless of the restrictions during the lockdown. The simulation results of our model are shown in Fig. 8. When *T*_0_ = 5, the peak of the proportion of the infected people is 5.8%, 87.7% of the residents in the whole community are uninfected, the recovered population accounted for 6.4%, and the mortality rate of the whole community is only 0.1%. However, as the start time of *T*_0_ is delayed, the spread of the COVID-19 in the community will become severe. When *T*_0_ = 10, the peak of the proportion of the infected people reaches 17.2%, the proportion of the uninfected residents drops to 55.9%, and the mortality rate is 1.8%. When the lockdown is implemented from the 15^*th*^ day, the proportion of the infected population suddenly rises to 84.6% on the 35^*th*^ day. By the end of the simulation, only 12.1% of residents in the community are uninfected, and the proportion of the deaths rises to 4.9%. When *T*_0_ is 20 and 30, the COVID-19 has already spread widely before the lockdown and accumulated enough numbers of the infected population to an outbreak. Implementing the lockdown measures is not helpful, especially when the lockdown measures are implemented on the 30^*th*^ day. The situation is equivalent to without lockdown. Only 2% of residents are not infected, and the proportion of deaths is 5.7%.

Figure 8 shows the number of infected people increasing exponentially from day 10 to 40 with different circumstances. This phenomenon reveals that *T*_0_ = 10 is a critical time. Lockdown before the 10^*th*^ day will suppress the spread of the epidemic effectively. If the lockdown is implemented later than the 10^*th*^ day, it will fail to control the spread of the COVID-19. At the same time, it’s important to note that in both cases of *T*_0_ = 5 and *T*_0_ = 10, the curves of the proportion of the infected people are zigzags after the implementation of the lockdown, which means the mutual infection between families in the same unit is possible. Some residents in the community who can move freely after the lockdown, which will lead to the breakthrough of the lockdown. This result emphasizes the necessity of strict restriction of population mobility. It will become the breakthrough for the spread of the COVID-19 even if only a small number of people move.

#### The impact of the timing of removing the lockdown measures (*T*_1_) on the control effect

**Fig. 9:**
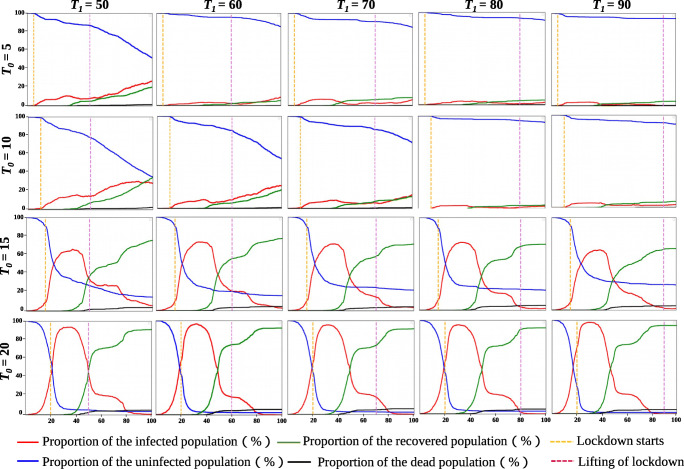
A schematic diagram of the spread of the epidemic in the community when the lockdown is lifted on the 50^*th*^, 60^*th*^, 70^*th*^, 80^*th*^, and 90^*th*^ days. *T*_0_ denotes the time when the lockdown starts, and *T*_1_ denotes the time when the lockdown is lifted

We use the settings from the previous experiment and introduce a mechanism for removing the lockdown on *T*_1_. The impact on removing the lockdown on the 50^*th*^ day, 60^*th*^ day, 70^*th*^ day, 80^*th*^ day and, 90^*th*^ day are respectively tested. Part of the results is shown in Fig. 9. When *T*_0_ is 30 or greater, the spread of the epidemic in the community is almost equivalent to the situation without lockdown. When *T*_0_ = 5, as *T*_1_ gradually increases from 50 to 90, the proportion of uninfected population in the community increases from 51% to 92.6%, and the peak number of infected people drop from 28.2% to 4.9%. The same result is further verified when *T*_0_ = 10. The proportion of the uninfected population is 34.4% when the lockdown measures are lifted on the 50^*th*^ day and 89.6% when the lockdown measures are lifted on the 90^*th*^ day. The number of infected people also shows a gradual downward trend. When *T*_0_ is 15 or 20, we find that results do not correlate with the value of *T*_1_, which are highly similar to each other. This means that once the lockdown is later than a critical time, the spread of the COVID-19 is almost unaffected by the time when the lockdown is lifted.

We discuss this in two situations. When *T*_0_ is less than or equal to 10, the effect of lockdown mainly depends on when the lockdown is lifted (*T*_1_). The benefits of early lockdown will be invalidated after it is lifted. Once the lockdown is lifted, the proportion of the infected people will begin to rise. The sooner the lockdown is lifted, the more complex the situation of the COVID-19 will be. When *T*_0_ is greater than or equal to 15, the effect of lockdown mainly depends on *T*_0_. The later the lockdown starts, the worse the spread of the COVID-19. Since the COVID-19 has a wider spread before lockdown, people have already gathered around the living units, although the lockdown has restricted the movement of residents to a smaller extent. If the proportion of infected residents breaks through a critical point, the lockdown will only have a weak control effect. In the worst case, the result will even be equivalent to the situation without lockdown measures.

### Model validation

Hunter et al. [15] proposed several ways to validate a simulation model. Many references mentioned above also confirm the rationality of these verifications. The obvious way is to use real data as the baseline. The effectiveness of the model is verified by comparing simulation results with real data. Parameter settings and assumptions can be checked through residual analysis. Our verification requires a variety of real data, including the real size of the community, the houseunits and their distribution, the number of residents, the number of infections/recoveries/deaths per day, and other necessary data. However, the government and related agencies currently do not release community-level data for COVID-19. Therefore, we derive community-level data from macro statistics released by WHO and NHC. In the absence of necessary real data, this verification is temporarily unavailable.

The other verification is to compare with the proven models. The SI model, SIR model, and their variants [19, 24] are suitable options. Although these compartmental models cannot provide simulation results on the agent level, they can still be used as control experiments for the population level. We take this verification and further supplement the comparative experiment.

**Fig. 10:**
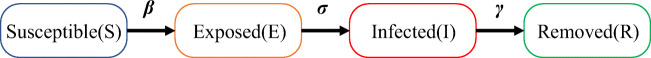
A schematic of the SEIR model

Compartmental models simplify the mathematical modeling of infectious diseases. Some agent-based models are compared with compartmental models such as SIR because they have the same state settings (Susceptible (S), Infected (I), Removed (R) et al.). Considering the settings of our model, the most suitable compartment model is the SEIR model (Fig. 10). The population is assigned to compartments with four labels, including Susceptible (S), Exposed (E), Infected (I), and Removed (R). In the COVID19_ALPS model, the counts for recovered and dead are kept separate, while in the SEIR model, these two categories are combined as removed (final state without contagious). Under normal circumstances, the SEIR model does not consider vital dynamics, which means no births or deaths in a closed population (the total population N is a constant). The SEIR model is defined by (3), where *N* = *S* + *E* + *I* + *R*. In particular, the exposed agents are not contagious in the SEIR model.

3 $$ \begin{aligned} &\frac{dS}{dt}=-\frac{\beta SI}{N} \\ &\frac{dE}{dt}=\frac{\beta SI}{N}-\sigma E \\ &\frac{dI}{dt}=\sigma E-\gamma I \\ &\frac{dR}{dt}=\gamma I \end{aligned}  $$

SEIR model cannot achieve lockdown, and we use the simulation results without lockdown as a comparison. The parameter settings are shown in the Table 4.

**Table 4:** The parameter settings in the SEIR model and the COVID19_ALPS model

Parameters	COVID19_ALPS	SEIR	Value
The total population	N	N	1,000
Prob of being infected	*P*_*I*_	*β*	0.05
Prob of morbidity	*P*_*m*_ + *P*_*c*_ + *P*_*a*_	*σ*	1
Prob of being recovered	*P*_*m*−*R*_ / *P*_*c*−*R*_	-	0.95/0.86
Prob of being removed	-	*γ*	0.95
Houseunits	h	-	16
Simulation duration	T	T	100 days

**Fig. 11:**
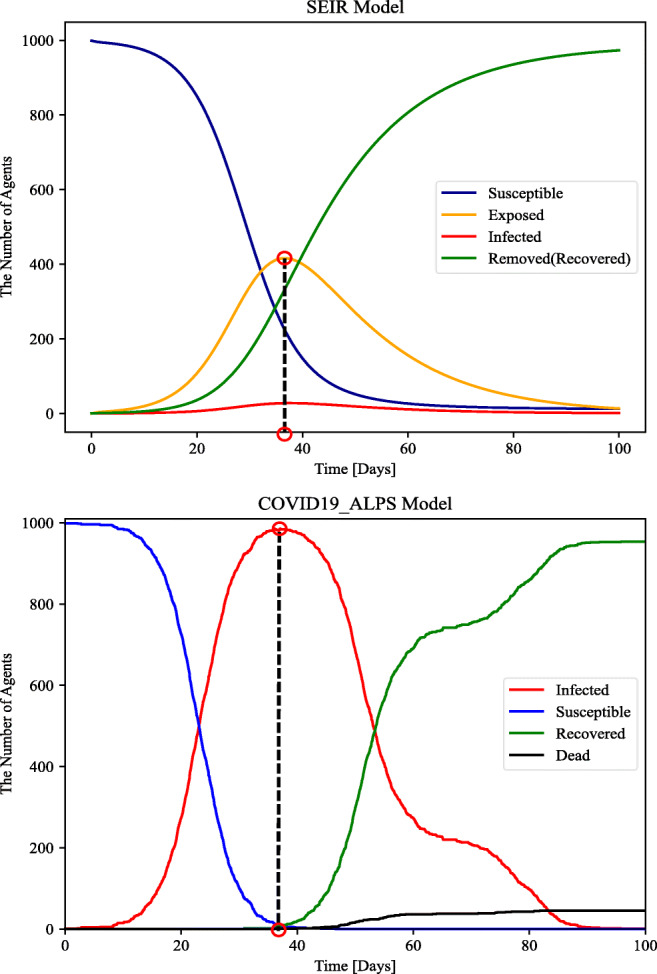
The comparative experiment between the SEIR model and COVID19_ALPS model

The COVID19_ALPS model and the SEIR model are essentially two different types of models, but the results (shown in Fig. 11) of the SEIR model verify the effectiveness of the COVID19_ALPS model in three aspects.

1. The number of infections will peak before the 40^*th*^ day. Since the exposed agents in the SEIR model are not contagious, the number of infected agents is far less than the COVID19_ALPS model.
2. In an ideal state without a lockdown, the spread of COVID-19 will reach its final state (all agents have recovered or died) before the 100^*th*^ day.
3. The number of susceptible agents decreased exponentially before the 40^*th*^ day.

In addition, we consider that comparison with recent related work can further verify the effectiveness of our model. References [23] and [25] use agent-based models to research COVID-19, and their results are worth learning. Among them, reference [23] proposes an agent-based model that simulates the spread

of COVID-19 among the inhabitants of a city. We tentatively set the parameters based on data from China to make the model run. The parameters are set as follows.

1. **tracing_percentage = 1.0 (100%)** In our research, it is assumed that all agents will be tracked.
2. **smartphone_owner_percentage = 1.0 (100%)** We do not consider anything about smartphones, setting this parameter to 100% can avoid experimental bias.
3. **quarantine_days = 14** “14” is a parameter setting that conforms to the reality in China.

**Fig. 12:**
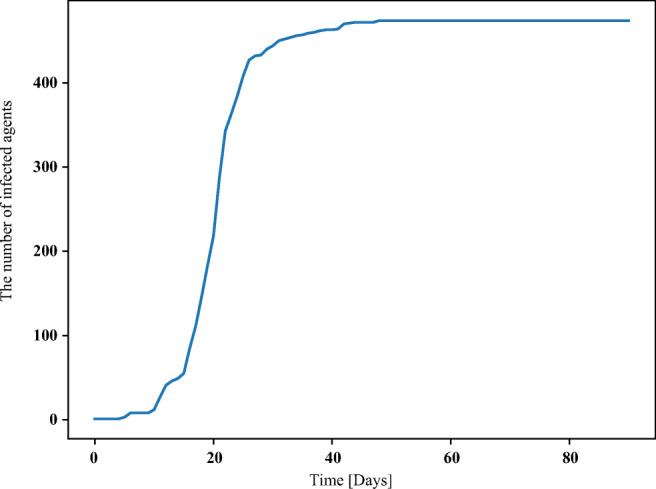
Simulation results of reference [23]

The result is shown in Fig. 12 This is a typical sigmoid growth curve similar to the natural growth curve of infected people in some cities. It can be confirmed in Figs. 14 and 15. Therefore, their model can simulate city-level data well, but there is no evidence that it can process community-level data. Combining the work of both will be interesting research in the future.

**Fig. 13:**
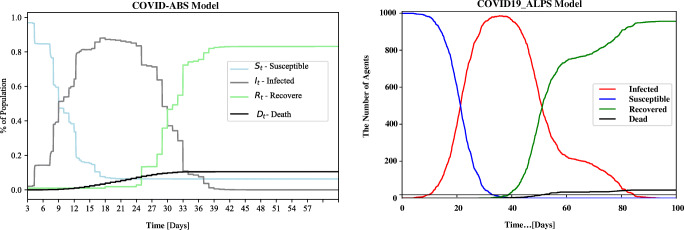
Simulation results without lockdown from COVID-ABS model (left) and COVID19_ALPS model (right)

The paper [25] proposes the COVID-ABS, a new SEIR agent-based model that aims to simulate the pandemic dynamics using a society of agents emulating people, business, and government. Due to the consideration of the economic factors, the simulation model of this work is very complicated and involves many parameters. Seven different scenarios of social distancing interventions were analyzed: (1) do nothing, (2) lockdown, (3) conditional lockdown, (4) vertical isolation, (5) partial isolation, (6) use of face masks, and (7) use of face masks together with 50% of adhesion to social isolation. Only Scenario (1) and Scenario (2) are relevant to our study. In Scenario (2), the author forced all agents to stop moving from *t* = 0 to T during the simulation. The experimental result is almost a straight line, which means that there are no new infections, and only the initial infected agents gradually recover or died. This result is in line with the facts, but it is different from our work. Therefore, Scenario (1) is the only thing we need to discuss. The results shown in Fig. 13 prove the effectiveness of our model. The curve shapes of the simulation results of both of us are highly similar. Since COVID-ABS considers complicated schedules, their result curve is jagged. Our model can achieve the same result with a more straightforward design, which is an advantage that cannot be ignored.

In a nutshell, it is still a complex problem to verify the agent-based model. Most researchers put forward agent-based simulation based on some special conditions to analyze the influence of variables in an ideal environment. This leads to the lack of suitable real data for these studies, and it is not easy to compare different simulations with each other. Nevertheless, the agent-based simulation model provides valuable forward-looking explorations for many real-world scenarios, important for governments and their staff.

### Comparison with real data in Wuhan and other cities in China

COVID19_ALPS has constructed an enclosed environment. The typical real case is the early stage of the COVID-19 in Wuhan, China. The local government of Wuhan closed all types of exits on January 23, 2020. The lockdown on Wuhan lasted for 76 days until April 8, 2020, and was gradually lifted and resumed. The lockdown of communities was implemented on February 10, 2020. Therefore, Wuhan can be considered as a large-scale closed community, which well matches the assumptions of the COVID19_ALPS model. January 23 is regarded as the starting time of the simulation in our model. The mandatory homestay implemented on February 10 means *T*_0_ = 18. The lockdown measures were lifted on April 8, which is considered as *T*_1_. The cut-off time is May 1 and the length of *T* is 100 days.

**Fig. 14:**
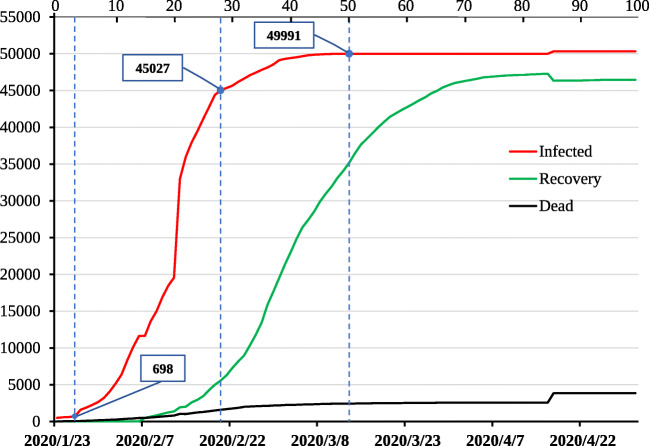
Daily epidemic in Wuhan from January 23, 2020 to May 1, 2020

It can be seen that the time of Wuhan’s lockdown of communities *T*_0_ is 18, which is larger than the critical value *T*_0_ = 10. Wuhan had 495 confirmed patients on January 23, which was larger than the COVID19_ALPS simulation. Therefore, the outbreak in Wuhan came earlier and lasted longer. Combining the COVID19_ALPS model and the real situation in Wuhan, we believe that the COVID-19 in Wuhan would show an exponential growth trend from the 5^*th*^ day to 30^*th*^ day, and the number of infections would reach the maximum near the 45^*th*^ day. The epidemic data in Wuhan confirms this conclusion, which is shown in Fig. 14. From January 26 to February 20, the number of people infected in Wuhan rose rapidly from 698 to 45,027. Subsequently, the growth of infections slowed down. On March 13, the number of infections reached 49,991. The growth of COVID-19 almost stagnated since then. Therefore, March 13 can be regarded as a peak of infection in the Wuhan epidemic, and 50 days have passed since January 23. We need to point out that the number of infected, cured, and deaths in Wuhan on April 18 in Fig. 14 changed slightly. This is not due to the rebound of COVID-19 after the lockdown measures were lifted. It is because of the data correction carried out by the local government.

**Fig. 15:**
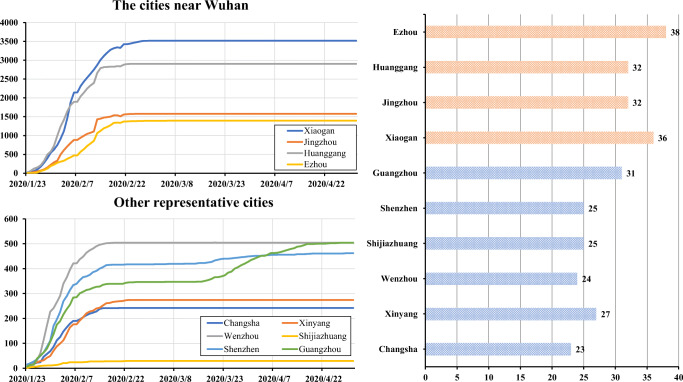
Daily epidemic in 10 cities from January 23, 2020, to May 1, 2020. (Left) and the length of time when the infections reach the maximum (Right)

In addition, we count the number of COVID-19 infections in ten cities during the same period. These cities are divided into two types: those close to Wuhan and other representative cities. The former includes Ezhou, Huanggang, Jingzhou, and Xiaogan. In Fig. 15 we show the changing process of the number of infections in these ten cities from January 23, 2020, to May 1, 2020. On the right side of the figure, we count the length of time between January 23, 2020, when the infected person reaches the maximum. The orange part represents the four cities close to Wuhan, and their data is greater than 30 days, which means that the spread of COVID-19 in these cities is severe. The remaining cities are far away from Wuhan, and the spread of COVID-19 is restricted more quickly. What needs to be pointed out is that Shenzhen and Guangzhou, as port cities, have experienced a second increase in the number of infected people. Our model’s judgment on the spread of COVID-19 has also been verified on the data of these cities.

In summary, the COVID19_ALPS model proposed in this research can be well adapted to real scenarios. The model can be used as a reference for epidemic prevention and control. It will help decision-makers understand the growth situation of the epidemic and the timing of implementing or lifting lockdown measures.

## Conclusion

This study has performed a quantitative evaluation on the effectiveness of the community lockdown through the COVID-19_ALPS model. We analyze the factors that affect the spread of COVID-19, including population mobility, community scale, the timings of implementing and lifting lockdown measures, etc. The appropriate parameter settings and the succinct assumptions make the COVID19_ALPS model efficient in simulation. The comparison with real data verifies the effectiveness of this model. The results reveal that:

1. Controlling population mobility is a necessary means of epidemic prevention. A tiny number of floating populations will cause the epidemic prevention to fall short of expectations.
2. Implementing a lockdown earlier will significantly limit the spread of the epidemic. Moreover, once the lockdown measures are implemented, they should not be lifted prematurely.
3. The movement of the population must be gradually restored when the situation is preventable and controllable.

However, there are still some limitations. The course of COVID-19 patients in the real world is far more complicated than hypothetical conditions. Patients in different disease states have mutual conversion. Community boundaries are usually not regular rectangles, and the distribution of residential units is not uniform, and so on. In particular, it should be pointed out that the assessment obtained by COVID19_ALPS is relatively conservative. In the future, more settings can be incorporated to make the model closer to the real world.

## Data Availability

Data sharing is not applicable to this work as no new data were created in this simulation study.

## Footnotes

1 http://www.nhc.gov.cn/jkj/s3578/202002/87fd92510d094e4b9bad597608f5cc2c.shtml?from=timeline&isappinstalled=0

2 http://www.nhc.gov.cn/

3 https://www.who.int/zh/emergencies/diseases/novel-coronavirus-2019/technical-guidancehttps://www.who.int/zh/emergencies/diseases/novel-coronavirus-2019/technical-guidance

4 http://www.mohurd.gov.cn/wjfb/201811/t20181130_238590.html

## Code Availability

The codes for the current study are available from the corresponding author on reasonable request.
